# Mitochondrial genetic effects on latent class variables associated with susceptibility to alcoholism

**DOI:** 10.1186/1471-2156-6-S1-S158

**Published:** 2005-12-30

**Authors:** Loren R Lease, Deidre A Winnier, Jeff T Williams, Thomas D Dyer, Laura Almasy, Michael C Mahaney

**Affiliations:** 1 Youngstown State University, Department of Sociology & Anthropology, One University Plaza, Youngstown, OH 44555, USA; 2 Department of Genetics, Southwest Foundation for Biomedical Research, San Antonio, TX, USA; 3 Southwest National Primate Research Center, Southwest Foundation for Biomedical Research, San Antonio, TX 78245, USA

## Abstract

We report the results of statistical genetic analyses of data from the Collaborative Study on the Genetics of Alcoholism prepared for the Genetic Analysis Workshop 14 to detect and characterize maternally inherited mitochondrial genetic effects on variation in latent class psychiatric/behavioral variables employed in the diagnosis of alcoholism. Using published extensions to variance decomposition methods for statistical genetic analysis of continuous and discrete traits we: 1) estimated the proportion of the variance in each trait due to the effects of mitochondrial DNA (mtDNA), 2) tested for pleiotropy, both mitochondrial genetic and residual additive genetic, between trait pairs, and 3) evaluated whether the simultaneous estimation of mitochondrial genetic effects on these traits improves our ability to detect and localize quantitative trait loci (QTL) in the nuclear genome. After correction for multiple testing, we find significant (*p *< 0.009) mitochondrial genetic contributions to the variance for two latent class variables. Although we do detect significant residual additive genetic correlations between the two traits, there is no evidence of a residual mitochondrial genetic correlation between them. Evidence for autosomal QTL for these traits is improved when linkage screens are conditioned on significant mitochondrial genetic effects. We conclude that mitochondrial genes may contribute to variation in some latent class psychiatric/behavioral variables associated with alcoholism.

## Background

A number of studies have report associations between mtDNA variation and chronic alcoholism. However, the deletions, insertions, modifications, and other rearrangements described in these studies represent damage done to mtDNA in the liver, white blood cells, and elsewhere as a consequence of oxidative stress associated with chronic ethanol consumption [1,2]. Only a small number of studies have detected associations between mtDNA polymorphisms and psychiatric disorders and syndromes that overlap those of chronic alcoholism [3,4]. These observations, plus the possibility that heritable mtDNA mutations could influence susceptibility to alcohol-induced oxidative stress damage, motivate our current study in which we analyze data from the Collaborative Study of the Genetics of Alcoholism (COGA) [5] to detect and characterize mitochondrial genetic effects on variation in latent class psychiatric/behavioral variables.

## Methods

### Data

The COGA dataset for the Genetic Analysis Workshop 14 includes 1,614 individuals, 788 females and 826 males, aged 17–91 years (mean = 40.09 ± 15.2 years), in 143 pedigrees of varying size and complexity. Families were recruited into COGA on the basis of a positive diagnosis of alcoholism (DSM-III-R (later, DSM-IV) and Feighner criteria) for a focal proband and 2 first degree relatives.

We analyzed data on the 14 latent class variables assessed in 1,181 to 1,388 of the COGA family members. The majority of these latent class variables were measured by COGA researchers on either a dichotomous (no = 1 and yes = 2; recoded as no = 0 and yes = 1 in our analyses) or ordinal scale (0, 1, 2, ..., n). These variables include: persistent desire to stop drinking (DESIRE), morning drinking (MORNING), craving for a drink (CRAVING), binge drinking (BINGE), narrowing of drinking repertoire (NARROW), gave up other activities to drink (GAVE UP), experience three or more blackouts (BLACKOUT), experienced withdrawal symptoms (WITHDRAWAL), experience physical health problems as a result of drinking (HEALTH), and experienced emotional/psychological problems as a result of drinking (PSYCHOL). By combining the 2 non-zero categories for the ordinal scale variable, spent so much time drinking there was little time for anything else (SPENT), we converted it to a dichotomous trait. We also analyzed the 2 continuous scale traits: maximum number of drinks in a 24 hour period (MAX DRINK) and the number of cigarette packs per day for one year (CIGPKYRS). In linkage analyses reported here we used genotype data at 315 microsatellite marker loci distributed across the 23 chromosomes in 1,376 individuals.

### Variance decomposition

Pedigree, phenotype, and genotype data were managed using PEDSYS [6] routines. We used a maximum likelihood based variance decomposition approach and extensions to this approach, implemented in SOLAR [7], to conduct all statistical genetic analyses. For basic statistical genetic analyses of continuously distributed traits, this approach models the phenotypic covariance as the sum of the additive genetic and random environmental covariances. We simultaneously estimated for each trait its mean, the mean effects of age and sex, a residual heritability (i.e., h^2 ^= ), and residual environmental standard deviation. We extended this basic approach to discrete traits using a multivariate normal threshold model in which the discrete trait is assumed to be determined by a threshold process acting on an underlying, continuous liability distribution [8]. To detect and estimate the effects of a maternally inherited mitochondrial effect on these traits, we employed the approach in Czerwinski et al. [9] in which the within-pedigree covariance matrix is modified by introducing an additional variance component, , and its structuring matrix M. M specifies the mitochondrial relationships in the pedigree that share a maternal line of descent [9]; thus matrix element *m*_*ij *_= 1 if individuals *i*,*j *are members of the same maternal lineage and *m*_*ij *_= 0 otherwise [10,11]. Aspects of mitochondrial inheritance such as heteroplasmy, threshold effects, phenotypic heterogeneity, and mtDNA/nuclear DNA interactions were not specifically modeled [11].

### Bivariate variance decomposition

We tested for possible mitochondrial genetic pleiotropy between any pairs of traits for which we detected a significant mitochondrial genetic component using a bivariate extension to the univariate variance components approach. This extension allows for the simultaneous estimation of all parameters estimated in the univariate analyses, plus the estimation of a residual additive genetic correlation, a mitochondrial genetic correlation, and a random environmental correlation between pairs of traits. For bivariate analyses of continuous and discrete trait pairings, we employed the method introduced by Williams et al. [12] to allow the liability of the discrete trait to be correlated with the quantitative trait.

### Adjustment for multiple tests

We used genetic correlations between all 78 latent trait pairs in a modified Bonferroni correction procedure to control for false-positive detection of mitochondrial genetic components to the variance in these 14 traits. To obtain an adjusted *p*-value consonant with α = 0.05, we divided 0.05 by 1 + (N - 1)(1 - |ρ_G_|), where N is the number of phenotypes, and |ρ_G_| is the mean of the absolute value of the genetic correlations.

### Linkage

We conducted a series of multipoint, whole genome linkage screens for each latent class variable exhibiting a significant (corrected *p *< 0.009) mitochondrial effect to detect evidence for quantitative trait loci (QTL) in nuclear chromosomal regions (only marker-specific linkage analyses were conducted with X-chromosome markers). Second, we performed locus-specific linkage analyses at the multipoint peaks providing the best evidence for a QTL for each trait to obtain an estimate of the improvement in the LOD score conditional on the mitochondrial genetic component.

### Likelihood ratio tests

Significance of all parameters was determined by likelihood ratio tests in which the likelihood of a more general model in which all parameters are estimated was compared to a nested restricted model in which some parameter values are constrained. The test statistic, twice the difference in the log likelihoods of the two models, was asymptotically distributed approximately as a χ^2 ^variate with degrees of freedom equal to the difference in the numbers of estimated parameters in the models. However, when a parameter value was constrained to the boundary of the parameter space (e.g., h^2 ^= 0), the test statistic was distributed as a 1/2:1/2 mixture of χ^2 ^and a point mass of zero [13]. To control for the overall false-positive rate given the finite marker locus density in the COGA microsatellite genome linkage map, we estimated genome-wide *p*-values by means of a method suggested by Feingold et al. [14]

## Results

Table 1 provides maximum likelihood estimates (MLEs) for components of the variance from a restricted model in which the proportion of the residual phenotypic variance due to the mitochondrial genetic component is constrained to zero (left side) and the more general model in which the effect of mitochondrial genetic component is estimated. A nominally significant mitochondrial genetic component (*p*(h^2^_mt _= 0) ≤ 0.05) is detected for three traits: DESIRE, CIGPKYRS, and CRAVING but, after correction for multiple testing, only the CIGPKYRS and CRAVING remain significant (*p *< 0.009; 78 pair-wise genetic correlations |mean ρ_G_| = 0.64). The mitochondrial genetic component accounts for 14% and 19% of the residual phenotypic variance and 77% and 65% of the additive genetic variance in these two traits, respectively.

Bivariate genetic analysis of the two traits identified above yielded the following correlation estimates when no mitochondrial genetic component was included in the model: ρ_G _= 0.06 ± 0.17, ρ_E _= 0.24 ± 0.07, and ρ_P _= 0.06. When a mitochondrial genetic component was included in the bivariate model for the traits, we detected a significant residual additive genetic correlation (ρ_G _= 1.00 ± 0.11), but the estimated mitochondrial genetic correlation between these two traits (ρ_M_= -0.36 ± 0.34) was not significantly different from zero (*p *> 0.05). The MLE for the residual additive genetic correlation between these two latent class variables was indicative of complete, or nearly complete, pleiotropy. Given the standard errors around the estimate, 60% to 100% of the residual additive genetic variance in each of these traits was due to the effects of genes that also influence the other trait. The residual correlation due to the shared effects of unmeasured environmental factors (i.e., due to the effects of covariates not included in the models, measurement errors, and/or non-additive genetic components) was significantly different from zero (ρ_E _= 0.20 ± 0.05, *p *< 0.05).

Our univariate multipoint linkage screens detected no significant evidence for a QTL influencing these two latent class variables on any of the autosomes. The best evidence for an autosomal QTL for each of these three traits is presented in Table 2. Subsequent maximization of these linkage models at the locations of their multipoint LOD peaks, conditional on the mitochondrial genetic component, raised statistical support for each QTL. None achieved genome-wide significance at the α = 0.05 level (LOD = 2.77).

## Discussion

The magnitude of the mitochondrial genetic effect on the phenotypic variance of the two latent class traits is likely small. However, mitochondrial genetic effects do account for a substantive proportion of the additive genetic variance in each trait and this proportion may be of biological, as well as statistical, significance.

Possible confounding of mitochondrial genetic and non-mitochondrial maternal effects (both genetic and environmental) is a valid concern; the range of pedigree size and complexity in the COGA families may mitigate this concern. We also believe the following observations provide circumstantial evidence that such confounding has not occurred. 1) Addition of the mitochondrial genetic component results in diminution of the residual additive genetic, rather than the environmental, component of the variance. 2) Re-analyses of these data incorporating the following surrogates for maternal effects as covariates, do not eliminate the mitochondrial genetic effect: maternal age at birth and household effects (unpublished data).

Our analyses of these data cannot identify the genes, gene products that may be involved, or the mechanisms by which they operate. However, the lack of a significant mitochondrial genetic correlation between the two traits tentatively suggests that multiple mtDNA variants, either in the same gene or in different genes, are responsible for the mitochondrial effects.

Lastly, our analyses offer tentative support for inclusion of significant mitochondrial genetic components in variance components models used in linkage screens. In each case, the LOD scores for non-significant multipoint peaks increased, although not quite to the level of genome-wide significance given the finite marker density of the linkage map and the mean recombination frequency in the COGA pedigrees.

## Conclusion

We detect evidence of mitochondrial genetic variation in two latent class phenotypes associated with susceptibility to alcoholism in data from the COGA families. To our knowledge, this is the first report of such an effect on any alcoholism-related traits.

## Abbreviations

COGA: Collaborative Study on the Genetics of Alcoholism

MLE: Maximum likelihood estimate

mtDNA: Mitochondrial DNA

QTL: Quantitative trait locus

## Authors' contributions

LRL and DAW conducted the analyses and interpreted the results of the univariate statistical genetic analyses and were responsible for writing and editing the final manuscript. JTW, instrumental in the development and implementation of both the mixed continuous-discrete bivariate analyses and the method for detecting the mitochondrial genetic component, provided assistance with analyses and interpretation of results. TDD and LA coordinated preparation of the COGA data for investigators at SFBR. MCM was responsible for funding, supervising, writing and editing the initial manuscript, and conducting and interpreting the bivariate analyses. All authors have read and approved this manuscript.

## Figures and Tables

**Table 1:** Proportions of the residual phenotypic variance in latent class variables

Trait	Restricted (h^2^_mt _= 0)			Unrestricted (h^2^_mt _≠ 0)				
		
	h^2^	e^2^	c^2a^	e^2^	h^2^	h^2^_mt_	c^2^	*p*(h^2^_mt _= 0)
MAX DRINK	0.16	0.84	0.21	0.85	0.12	0.03	0.21	0.10
SMOKER	0.43	0.57	n/a	0.75	0.10	0.14	n/a	0.15
DESIRE	0.47	0.53	n/a	0.55	0.35	0.10	n/a	0.05
MORNING	0.18	0.82	n/a	0.84	0.12	0.04	n/a	0.29
CIGPKYRS	0.17	0.83	0.21	0.82	0.04	0.14	0.21	0.002
CRAVING	0.29	0.71	n/a	0.71	0.10	0.19	n/a	0.004
BINGE	0.06	0.94	n/a	0.96	0.00	0.03	n/a	0.28
SPENT	0.07	0.93	0.01	0.94	0.05	0.01	0.01	0.31
NARROW	0.34	0.66	n/a	0.68	0.26	0.06	n/a	0.23
GAVE UP	0.26	0.74	n/a	0.82	0.07	0.11	n/a	0.07
BLACKOUTS	0.38	0.62	n/a	0.62	0.36	0.02	n/a	0.38
WITHDRAWAL	0.20	0.80	n/a	0.84	0.11	0.05	n/a	0.28
HEALTH	0.26	0.74	n/a	0.76	0.23	0.01	n/a	1.00
PSYCHO	0.36	0.64	n/a	0.64	0.34	0.01	n/a	0.42

^a^Proportion of total phenotypic variance due to covariates (c^2^), additive genetic effects (h^2^), mitochondrial genetic effects (h^2^_mt_), and random environmental factors (e^2^).

**Table 2:** LOD scores from linkage screens with and without mitochondrial effects

Trait	h^2^_mt _= 0		h^2^_mt _≠ 0			QTL Location		
			
	LOD	Genome-wide *p*	LOD	Genome-wide *p*	Locus-specific *p*	cM from pter	pter STR locus	qter STR locus
CIGPKYRS	1.68	0.76	2.67	0.06	0.00045	158	D2S1328	D2S1334
CRAVING	1.41	1.49	2.21	0.20	0.00014	258	D3S1311	3-qter
